# Supplementary material for: Could Cratylia argentea replace Tifton 85 hay on growing and finishing lamb diets in tropical areas?
Source: PLoS One. 2023 Dec 11;18(12):e0295510. doi: 10.1371/journal.pone.0295510 (PMC10712867; doi:10.1371/journal.pone.0295510)
Supplement: Attachment — Submitted filename: PONE-D-23-03057_reviewer.pdf [file pone.0295510.s002.pdf]

PLOS ONE

Could Cratylia argentea replace Tifton 85 hay on finishing lamb diets in tropical areas?

--Manuscript Draft--

**Manuscript Number:**	PONE-D-23-03057
**Article Type:**	Research Article
**Full Title:**	Could Cratylia argentea replace Tifton 85 hay on finishing lamb diets in tropical areas?
**Short Title:**	Cratylia as a potential replacement to Tifton 85 hay on finishing lamb diets
**Corresponding Author:**	Elaine Cristina Teixeira, Ph.D. student<br>Universidade Federal de Sao Joao del-Rei<br>Sete Lagoas, MG BRAZIL
**Keywords:**	forage conservation, legume digestibility, ruminant feeding, shrub legume, Cratylia argentea
**Abstract:**	Legumes shrubs such as Cratylia argentea have an ability to thrive in environments with low water availability and poor soil. On the other hand, forage grasses such as Tifton 85 have a greater demand for inputs to be productive. The objective of this study was to evaluate the performance of finishing Lacaune lambs fed Cratylia argentea hay as an alternative to Tifton 85 (Cynodon spp). Twenty-four Lacaune lambs aged between five and six months (average body weight [BW] 21.50 ± 3.38 kg) were arranged in a split-plot randomized block design. The plots consisted of different Cratylia to Tifton 85 hay proportions (0, 20%, 40%, or 100%, dry matter [DM] basis) as a roughage replacement in the total diet. The subplots represented two evaluation times, entitled “initial period” and “final period”, which consisted of the early seven days of total feces and urine collection, and the last seven days of the experiment, respectively. The lambs were blocked by weight with six replicates per treatment. The results show that the level of Tifton 85 replacement for Cratylia hay in the roughage portion of the lamb diet did not influence (P > 0.05) weight gain (WG), dry matter intake or dry matter digestibility; feed conversion, feed efficiency; and the evaluated nitrogen balance variables. The digestibility coefficient of neutral detergent fiber decreased linearly as Tifton 85 replacement for Cratylia level was increased, which probably happened due to the presence of highly lignified material within the Cratylia hay. However, the alternative legume maintained animal performance of Tifton 85. In conclusion, Cratylia hay can be recommended as a potential substitute for Tifton 85 hay, which requires greater inputs for the production. Cratylia may be considered a feeding strategy for livestock production, especially for smallholder livestock systems and regions with unfavorable soil and climate.
**Order of Authors:**	Elaine Cristina Teixeira, Ph.D. student
	Lucas Abreu, Ph.D. student
	Fernando de Souza
	Walter Matrangolo
	Luciano de Lima
	Karina da Silva
	Hemilly Menezes de Sa
	Ângela Maria Quintão Lana
**Additional Information:**	
**Question**	**Response**
**Financial Disclosure**<br><br>Enter a financial disclosure statement that describes the sources of funding for the work included in this submission. Review	This work was supported by the National Council for Scientific and Technological Development (CNPq, Brazil nº 140314/2019-9), State Agency for Research and Development of Minas Gerais (FAPEMIG, PPM-00639-18) and Coordination for the Improvement of Higher Education Personnel of Education (CAPES, Brazil nº 88887.667823/2022-00).

Powered by Editorial Manager® and ProduXion Manager® from Aries Systems Corporation

the submission guidelines for detailed requirements. View published research articles from *PLOS ONE* for specific examples.<br><br>This statement is required for submission and **will appear in the published article** if the submission is accepted. Please make sure it is accurate.<br><br>**Unfunded studies**<br>Enter: *The author(s) received no specific funding for this work.*<br><br>**Funded studies**<br>Enter a statement with the following details:<br>• Initials of the authors who received each award<br>• Grant numbers awarded to each author<br>• The full name of each funder<br>• URL of each funder website<br>• Did the sponsors or funders play any role in the study design, data collection and analysis, decision to publish, or preparation of the manuscript?<br>• **NO** - Include this sentence at the end of your statement: *The funders had no role in study design, data collection and analysis, decision to publish, or preparation of the manuscript.*<br>• **YES** - Specify the role(s) played.<br><br>* typeset	The funders had no role in study design, data collection and analysis, decision to publish, or preparation of the manuscript.
**Competing Interests**<br><br>Use the instructions below to enter a competing interest statement for this submission. On behalf of all authors, disclose any competing interests that could be perceived to bias this work—acknowledging all financial support and any other relevant financial or non-financial competing interests.<br><br>This statement is required for submission and **will appear in the published article** if the submission is accepted. Please make sure it is accurate and that any funding sources listed in your Funding Information later in the submission form are also	The authors have declared that no competing interests exist.

declared in your Financial Disclosure statement.<br><br>View published research articles from *PLOS ONE* for specific examples.<br><br>**NO authors have competing interests**<br><br>Enter: *The authors have declared that no competing interests exist.*<br><br>**Authors with competing interests**<br><br>Enter competing interest details beginning with this statement:<br><br>*I have read the journal's policy and the authors of this manuscript have the following competing interests: [insert competing interests here]*<br><br>* typeset	
**Ethics Statement**<br><br>Enter an ethics statement for this submission. This statement is required if the study involved:<br><br>• Human participants<br>• Human specimens or tissue<br>• Vertebrate animals or cephalopods<br>• Vertebrate embryos or tissues<br>• Field research<br><br>Write "N/A" if the submission does not require an ethics statement.<br><br>General guidance is provided below. Consult the submission guidelines for detailed instructions. **Make sure that all information entered here is included in the Methods section of the manuscript.**	Ethics approval: all experimental procedures were approved by the Ethics Committee on Animal Use (CEUA) at the Federal University of Minas Gerais (approval no. 192/2020).

**Format for specific study types**<br><br>**Human Subject Research (involving human participants and/or tissue)**<br>• Give the name of the institutional review board or ethics committee that approved the study<br>• Include the approval number and/or a statement indicating approval of this research<br>• Indicate the form of consent obtained (written/oral) or the reason that consent was not obtained (e.g. the data were analyzed anonymously)<br><br>**Animal Research (involving vertebrate animals, embryos or tissues)**<br>• Provide the name of the Institutional Animal Care and Use Committee (IACUC) or other relevant ethics board that reviewed the study protocol, and indicate whether they approved this research or granted a formal waiver of ethical approval<br>• Include an approval number if one was obtained<br>• If the study involved *non-human primates*, add *additional details* about animal welfare and steps taken to ameliorate suffering<br>• If anesthesia, euthanasia, or any kind of animal sacrifice is part of the study, include briefly which substances and/or methods were applied<br><br>**Field Research**<br><br>Include the following details if this study involves the collection of plant, animal, or other materials from a natural setting:<br>• Field permit number<br>• Name of the institution or relevant body that granted permission	
**Data Availability**<br><br>Authors are required to make all data underlying the findings described fully available, without restriction, and from the time of publication. PLOS allows rare exceptions to address legal and ethical concerns. See the PLOS Data Policy and FAQ for detailed information.	Yes - all data are fully available without restriction

A Data Availability Statement describing where the data can be found is required at submission. Your answers to this question constitute the Data Availability Statement and **will be published in the article**, if accepted.<br><br>**Important:** Stating 'data available on request from the author' is not sufficient. If your data are only available upon request, select 'No' for the first question and explain your exceptional situation in the text box.<br><br>Do the authors confirm that all data underlying the findings described in their manuscript are fully available without restriction?	
**Describe where the data may be found in full sentences. If you are copying our sample text, replace any instances of XXX with the appropriate details.**<br><br>• If the data are **held or will be held in a public repository**, include URLs, accession numbers or DOIs. If this information will only be available after acceptance, indicate this by ticking the box below. For example: *All XXX files are available from the XXX database (accession number(s) XXX, XXX.*).<br>• If the data are all contained **within the manuscript and/or Supporting Information files**, enter the following: *All relevant data are within the manuscript and its Supporting Information files.*<br>• If neither of these applies but you are able to provide **details of access elsewhere**, with or without limitations, please do so. For example:<br><br>*Data cannot be shared publicly because of [XXX]. Data are available from the XXX Institutional Data Access / Ethics Committee (contact via XXX) for researchers who meet the criteria for access to confidential data.*<br><br>*The data underlying the results presented in the study are available from (include the name of the third party*	All relevant data are within the manuscript and its Supporting Information files.

and contact information or URL).<br>• This text is appropriate if the data are owned by a third party and authors do not have permission to share the data.<br><br>* typeset	
Additional data availability information:	Tick here if your circumstances are not covered by the questions above and you need the journal’s help to make your data available.

Powered by Editorial Manager® and ProduXion Manager® from Aries Systems Corporation

Manuscript

Click here to access/download;Manuscript;Cratylia argentea as a replacement to Tifton 85 hay DEFLO.docx

Full title: **Could *Cratylia argentea* replace Tifton 85 hay on finishing lamb diets in tropical areas?**

Short title: Cratylia as a potential replacement to Tifton 85 hay on finishing lamb diets

Elaine Cristina Teixeira[1]*, Lucas Freires Abreu[2], Fernando Antônio de Souza[2], Walter José Rodrigues Matrangolo[3], Karina Toledo da Silva[4], Luciano Soares de Lima[2], Hemilly Cristina Menezes de Sa[2], Ângela Maria Quintão Lana[2].

[1] Departamento de Engenharia Florestal, Universidade Federal de São João del Rei, Sete Lagoas – MG, 35701-970, Brasil

[2] Departamento de Zootecnia, Escola de Veterinária, Universidade Federal de Minas Gerais, Belo Horizonte - MG, 31270-901, Brasil

[3] Empresa Brasileira de Pesquisa Agropecuária - Embrapa Milho e Sorgo, Sete Lagoas, MG 35701-970, Brasil

[4] Empresa de Pesquisa Agropecuária de Minas Gerais – EPAMIG Centro Oeste, Prudente de Morais, MG 35715-000, Brasil

*Corresponding author: elaineteixeira@ufsj.edu.br

Author contributions: **Elaine Cristina Teixeira**: Conceptualization, Investigation, Visualization, Writing – original draft, Project administration, **Lucas Freires Abreu**: Writing – review & editing, Investigation, **Fernando Antônio de Souza**: Formal analysis, Software, Data Curation, **Walter José Rodrigues Matrangolo**: Supervision, Resources**, Karina Toledo da Silva**: Supervision, Resources, **Luciano Soares de Lima**: Conceptualization, Methodology, **Hemilly Cristina Menezes de Sá**: Conceptualization, Methodology, **Ângela Maria Quintão Lana**: Supervision,

Conceptualization, Resources, Funding acquisition, Writing – review & editing. All authors have read and approved the manuscript.

Abstract

Legumes shrubs such as *Cratylia argentea* have an ability to thrive in environments with low water availability and poor soil. On the other hand, forage grasses such as Tifton 85 have a greater demand for inputs to be productive. The objective of this study was to evaluate the performance of finishing Lacaune lambs fed *Cratylia argentea* hay as an alternative to Tifton 85 (*Cynodon* spp). Twenty-four Lacaune lambs aged between five and six months (average body weight [BW] 21.50 ± 3.38 kg) were arranged in a split-plot randomized block design. The plots consisted of different Cratylia to Tifton 85 hay proportions (0, 20%, 40%, or 100%, dry matter [DM] basis) as a roughage replacement in the total diet. The subplots represented two evaluation times, entitled "initial period" and "final period", which consisted of the early seven days of total feces and urine collection, and the last seven days of the experiment, respectively. The lambs were blocked by weight with six replicates per treatment. The results show that the level of Tifton 85 replacement for Cratylia hay in the roughage portion of the lamb diet did not influence (P > 0.05) weight gain (WG), dry matter intake or dry matter digestibility; feed conversion, feed efficiency; and the evaluated nitrogen balance variables. The digestibility coefficient of neutral detergent fiber decreased linearly as Tifton 85 replacement for Cratylia level was increased, which probably happened due to the presence of highly lignified material within the Cratylia hay. However, the alternative legume maintained animal performance of Tifton 85. In conclusion, Cratylia hay can be recommended as a potential substitute for Tifton 85 hay, which requires greater inputs for the

production. Cratylia may be considered a feeding strategy for livestock production, especially for smallholder livestock systems and regions with unfavorable soil and climate.

Keywords: forage conservation, legume digestibility, ruminant feeding, shrub legume, *Cratylia argentea*

Introduction

Tifton 85 (*Cynodon* spp.) is one of the major grasses used for hay production in Brazil (Fonseca and Martuscello, 2010). Nonetheless, tropical grasses such as Tifton 85 can have its feed value considerably reduced due to lower digestibility and quality over water deficit periods. Moreover, its yield and use are linked to the adoption of management practices associated with the intensification of inputs use such as chemical fertilization (Jaramillo et al., 2018) and complementary irrigation.

The increased use of chemical fertilizers is linked to greater greenhouse gas (GHG) emissions and depletion of water resources, which happens due to nitrous oxide emissions and runoff, respectively (Snyder et al., 2009), thus, decreasing the sustainability of livestock systems. In the same context, a rise in nitrogen fertilizer costs have led nitrogen (N) fixing crops, such as legume shrubs, to become more attractive for producers, particularly of smallholder livestock systems.

Several leguminous shrubs and trees have been identified as feed alternatives for ruminants in tropical areas (Castro-Montoya and Dickhoefer, 2020; Wanapa et al., 2021). However, its use is yet to be adopted, possibly due to a lack of consensus on the most suitable species and how to incorporate them

into tropical production systems (Ates et al., 2018; Castro-Montoya and Dickhoefer, 2020).

Cratylia argentea (Cratylia) is a perennial legume shrub with a feeding potential for livestock in tropical areas. The species is native to the Brazilian Savanna (*Cerrado*) but is also found in other biomes such as the *Amazonian Rainforest* and *Caatinga* (Queiroz, 1991; Queiroz and Coradin, 1996). Although its Brazilian origin, Cratylia have previously been planted for livestock feeding on other countries such as Mexico (Valles-de la Mora et al., 2017), Costa Rica (Ibrahim et al., 2001) and Colombia (Hess et al., 2004).

Cratylia is very competitive not only in terms of mass production but largely because of its ability to symbiotically associate with nitrogen-fixing bacteria as well. It has great crude protein (CP) concentration and yielding potential, a symbiotic ability to fix atmospheric N (Matrangolo et al., 2018). Cratylia can maintain green leaves in critical periods of water deficit, thanks to its deep and spread root system (Matrangolo et al., 2018). In their initial growth phase, the Cratylia rootlets develop an external tissue similar to a cork, which helps to minimize water loss (Matrangolo et al., 2018). Those features allow Cratylia to keep growing during the dry season, despite being grazed or harvested (Argel and Lascano, 1998). The same authors reported that 30-40% of Cratylia’s yield may occur in the dry season, which makes a great feedstock to fill forage gaps in tropical regions. Another important characteristic of this legume is its tolerance to acidic soils with high aluminum saturation and low natural fertility (Maas, 1995; Luz et al., 2015). Dry biomass accumulation by this legume ranges from 14 to 21 t ha^{-1} yr^{-1}, without fertilizer application or supplemental water, as reported in previous research (Pizarro et al., 1996).

Several studies have demonstrated the benefits of including legume shrubs as an alternative for diet and performance improvement on ruminants (Hess et al., 2004; Valles-de la Mora et al., 2017; Herrera et al., 2021). Those studies found that these plants constitute a path towards sustainability, whether through direct grazing or for the enrichment of diets supplied in a trough (Ates et al., 2018; Castro-Montoya and Dickhoefer, 2020). Valles-de la Mora et al. (2017) evaluated the development of grazing heifers (F1 Holstein x Zebu) on *Brachiaria brizantha* cv. Toledo alone vs the association of Cratylia-*Brachiaria brizantha* Toledo and observed a superior weight gain on animals grazing the Cratylia treatment. The same study also observed CP levels of over 20% in Cratylia, which were very consistent throughout the experiment. Andersson et al. (2006) evaluated the nutritive value of 38 Cratylia accessions harvested at 8 weeks of regrowth and found a mean CP of 20,6% and 22% and in vitro digestible dry matter (IVDDM) of 64,1% and 64,9% for dry and rainy season, respectively. Ibrahim et al. (2001) evaluated the association of *Hyparrhenia rufa* pasture and fresh *Cratylia argentea* as a protein supplement with or without molasses (diluted in the water trough) for dual purpose cattle in the dry season of Costa Rica. The results showed an increase of IVDDM from 55.6 to 60.8% ($P < 0.01$) when Cratylia was associated with sprayed molasses. In addition, there was a 43% increase on intake of Cratylia, when treated with molasses, which also resulted in a greater intake (>13%; $P < 0.05$) of *H. rufa*.

With that being said, it is assumed that Cratylia hay might be an alternative to hay of forage grasses in the diet of finishing lambs. The objective of this study was to evaluate the performance of finishing lambs fed diets with increasing levels of Cratylia hay to replace Tifton 85 hay as the roughage source.

Materials and methods

Study area

The study was conducted at the Brazilian Agricultural Research Corporation – Embrapa Milho e Sorgo (Sete Lagoas, Minas Gerais, Brazil; 19º28′ S; 44º15′W, at 732 m altitude), where the Cratylia was planted for hay, and the Laboratory of Animal Metabolism and Calorimetry (LAMA/LACA) at the Veterinary School of the Federal University of Minas Gerais (EV-UFMG), Belo Horizonte, MG, where the lambs and infrastructure for the feeding experiment were located.

The unit where Cratylia was planted is in a region with a Cwa climate type according to the Köppen classification system, i.e., savanna climate, with dry winters and humid-rainy summers. Cratylia was transplanted from a growth chamber to a research site on March 28th 2013, and several trials were conducted in the area until 2019 when the current research began. Cratylia's shrubs were planted in an area of 105 m^2, spaced at 1 m between rows and a total of 126 plants, or 12,000 plants/ha^{-1}. The area size was determined based on the number of available Cratylia's sprigs found available with research peers before planting, since commercial seeds were not available at the time. The soil test for the area where Cratylia was planted for hay is described in Table 1. The Craltylia hay was produced entirely without the use of chemical fertilizers based on the potentially autogamous characteristic of the legume and to maintain the low production cost (Andersson et al., 2006). Tifton 85 hay was purchased from a local dealer and presented a dry matter (DM) of 91.25%, CP 13.2% and NDF 68.98%, which was used to formulate the feed mixture. The agronomical accessions for the harvested Cratylia hay are presented in Table 2.

Table 1. Soil test result (00 – 20 cm) and planting date of *Cratylia argentea*.

Planting date	pH	NO_3-N	P	K	OM
		---------------- (mg kg^{-1}) -----------------			(dag kg^{-1})
28/03/13	5.5	18	2.18	39.26	3.44

Soil pH was measured using a 1:1 soil:water, NO_3-N was measured using a 2M KCl extract, and plant available P and K were extracted using a Mehlich-3 solution (Mehlich, 1984).

Table 2. Agronomical accessions for the harvested *Cratylia argentea* hay.

	DM	CP	ADF	ADIN	NDF	NDIN
	93.2	19.6	41.6	0.492	58.4	2.28
sd	1.28	3.45	5.52	0.100	5.40	1.19

DM, dry matter; CP, crude protein; ADF, acid detergent fiber; ADIN, acid detergent insoluble nitrogen, NDF, neutral detergent fiber; NDIN, neutral detergent insoluble nitrogen; sd, standard deviation. n = 530

Cratylia hay and feed management

To establish a uniform regrowth, all Cratylia's plots were mowed in January 2019. The first harvest for data collection happened in April of the same year and was repeated every other 90 days, until April 2020 (Table 3), leaving a 25 cm stubble height (Pinzón and Mariño, 2010), without the selection of less lignified material. During the haymaking period, temperatures were between 17.74 and 29.24 °C, average rainfall was 885 mm, and relative humidity was 66.56% (NASA/POWER, 2022).

Table 3. *Cratylia argentea* harvest dates.

	2019	2020	
Harvest 1	04-02	Harvest 4	01-09
Harvest 2	07-10	Harvest 5	04-14
Harvest 3	10-07	-	-

To produce enough forage mass for the totality of animals, after each harvest, the produced hay was stored in raffia bags and packed in drums to avoid contamination, until the beginning of the feeding treatments at LAMA/LACA, which started in November 2020 and lasted until January 2020. All harvested

material was cut through a forage chopper then spread in a 10-cm layer on a cemented area for drying in the sun and turned frequently for drying to the point of hay, which happened when the Cratylia reached a DM content of 85%. The interval of exposure to the sun for the material to reach the point of hay varied between 48 and 72 h, depending on the weather conditions. During the night, or in case of rain, the material was covered with canvas to prevent moisture.

The Tifton 85 and *Cratylia* hay were ground to a particle size of 5 mm, homogenized, and then mixed with the other ingredients of the experimental diets according to the replacement levels (Table 4), as described by Lima (2016).

Table 4. Percentage and chemical composition of experimental diets.

Variable	**Replacement of Tifton 85 with *C. argentea***			
	**0%**	**20%**	**40%**	**100%**
Ingredient (g/kg DM)				
Cratylia argentea hay	-	100.0	200.0	441.3
Tifton 85 hay	549.9	425.4	300.7	-
Ground maize	237.2	266.4	295.6	366.1
Soybean meal	175.4	170.9	166.4	155.5
Mineral for sheep[1]	15.0	15.0	15.0	15.0
Limestone	10.5	10.5	10.8	11.3
Dicalcium phosphate	7.0	6.8	6.5	5.8
Sodium bicarbonate	5.0	5.0	5.0	5.0
Chemical composition				
Dry matter (g/kg)	908.4	913.2	903.3	903.0
Organic matter (g/kg DM)	919.9	923.1	922.4	923.9
Ash (g/kg DM)	80.1	76.9	77.6	76.1
Crude protein (g/kg DM)	178.4	188.8	180.7	182.2
Ether extract (g/kg DM)	24.9	13.0	16.3	17.7
Neutral detergent fiber (g/kg DM)	433.4	426.0	390.1	352.5
Total carbohydrates (g/kg DM)	716.6	721.4	725.4	724.0

Tortuga® (DSM) Mineral for sheep (content per kg of product): zinc - 3,800 mg; sodium - 147 g; manganese - 2,000 mg; cobalt - 15 mg; copper - 590 mg; sulfur - 18 g; selenium - 20 mg; iodine - 50 mg; chromium - 20 mg; molybdenum - 300 mg; calcium - 110 g; calcium (max.) - 135 g; fluorine (max.) - 870 mg; phosphorus - 87 g. DM – dry matter, NM – natural matter

Feeding trial

Twenty-four Lacaune lambs aged between five and six months (21.5 ± 3.38 kg) were divided into groups according to their initial weight (Table 5). The lambs were housed in individual metabolic cages (1.5 × 0.7 m) equipped with a feeding and water trough, and a system for separate collection of feces and urine, inside a covered masonry shed with an exhaust system at LAMA/LACA laboratories.

Table 5. Block distribution of lambs.

Block	Category	Mean weight (kg)	Standard deviation	Replicates per treatment
1	Light	15.9	0.580	1
2	Medium-light	20.6	0.970	2
3	Medium-heavy	23.5	0.640	2
4	Heavy	26.0	1.21	1

The total experimental period was 64 days, the first 21 of which were used as a period of adaptation to the experimental diets and cage management, followed by 43 days of intake and weight gain (WG) measurements. Digestibility trials were conducted in two periods, here entitled “initial” and “final”. The initial period consisted of 7 days of total collection of feces and urine, which began after the adaptation period (5 to 6 months, 26.8 ± 3.26 kg), and the final period began in the last 7 days of the experiment (6 to 7 months, 37.5 ± 3.26 kg).

Feed was supplied twice daily (08h00 and 14h00) for *ad libitum* intake (200 g/kg orts, as-fed basis). Supplied feed and orts were weighed daily on an electronic scale. Each of the diets was composed of different levels of Cratylia hay to replace Tifton 85 hay (Table 4). The diets were formulated to meet the requirements for finishing lambs with a DM intake of 4% BW and an average daily gain (ADG) of 200 g, thus, a 16% CP and total digestible nutrients (TDN) of 75%, following the NRC (2007). All diets were supplied as a total mixture ratio and adjusted daily allowing up to 20% orts.

Live weight was measured weekly from the beginning of the experimental period, in the morning period before the feed was supplied, using a platform scale. Initial live weight (ILW) was determined immediately after the end of the adaptation period, and final live weight (FLW) at 43 days of the experiment. The ADG was determined by the following equation: ADG = ((FLW– ILW)/(Number of days in the feedlot). Dry matter intake (DMI) was individually calculated as the difference between the amount of feed supplied and daily orts.

The digestibility trial involved total collection of feces and urine on two occasions: (1) in the first week and (2) in the last week of the experimental period. Samples of supplied feed, orts, and feces were analyzed for the dry matter (DM), ash, and nitrogen (N) contents according to AOAC (1990) and the neutral detergent fiber (NDF) content according to Van Soest et al. (1991). The total tract apparent digestibility coefficient (ADC) was determined as follows: ADC = ([OF – Intake – CF]/OF – Intake) × 100, which was proposed by Maynard et al. (1984), where AD = apparent digestibility; OF = offered feed [(offered feed amount in kg DM) x (offered nutrient content in % DM)/100]; Intake = orts [(removed orts feed

in kg DM) x (orts nutrient content in % DM)/100]; and CF = fecal output [(amount of collected feces in kg DM) x (collected feces content in % DM)/100].

Urine was collected in plastic containers containing a hydrochloric acid solution (6N HCl) to keep the urine pH below 2 and prevent the loss of nitrogen by volatilization. The total collected material was quantified, homogenized, and filtered through cotton gauze and a 10% sample was taken and stored at -18 °C. The nitrogen content of the sample was determined by the micro Kjeldahl method (Campos et al., 2004). Nitrogen balance was calculated according to Gentil et al. (2011).

Statistical analyses

The 24 lambs were allocated in a randomized block design with four blocks, where each block was randomly assigned with a dietary treatment of 0, 20, 40, and 100% Cratylia hay replacing Tifton 85 hay in the diet, and six replicates. The effect of treatments was submitted to an analysis of variance (ANOVA), with the following general model:

$$Yijkl = \mu + \tau i + \gamma k + eikl + \beta j + (\tau\beta)ij + \epsilon ijkl,$$

where Yijkl = value observed at the Tifton 85 replacement level for Cratylia hay level I, in block k, replicate l, and subplot j; μ = mean overall effect; τi = effect of Tifton 85 replacement level for Cratylia hay i; γk = effect of block k; ϵikl = error attributed to the plot Yikl (ϵik ~ N [μ, σ2]); βj = effect of collection period j; (τβ)ij = interaction between Tifton 85 replacement level for Cratylia hay i and collection period j; and ϵijkl = error attributed to subplot Yijk (ϵijkl ~ N [μ, σ2]).

For performance variables, the fits of polynomial regression models were tested to obtain the equation as a function of the Tifton 85 replacement levels for Cratylia hay or collection periods.

Analyses were carried out using R statistical analysis software (Core Team, 2021). For all analyses, the statistical assumptions of normality and homoscedasticity were evaluated by the Shapiro-Wilk and Bartlett tests, respectively. An effect was considered significant when the α error was less than 5%.

Results

Intake and digestibility of Cratylia hay

Dry matter intake did not differ ($P > 0.05$) in response to Tifton 85 replacement levels for Cratylia (Table 6). However, there was an increase of 12.5%, 17.64%, 18.75% and 16.67% in DMI with the respective increase in the proportions (0%, 20%, 30%, 40% and 100%) of Cratylia in the final collection period ($P < 0.05$), compared with the initial collection period.

Table 6. Intake and digestibility of dry matter and nutrients, feed conversion, and feed efficiency of feedlot Lacaune lambs fed diets containing different levels of *C. argentea* hay as a replacement Tifton 85 hay at two evaluation times.

Variable	0%		20%		40%		100%			*P*-value		
	Initial	Final	Initial	Final	Initial	Final	Initial	Final	SE	L	C	L×C
							Intake (kg/day)					
Dry matter	1.4	1.6	1.4	1.7	1.3	1.6	1.5	1.8	0.17	0.548	<0.001	0.617
							Digestibility (g/kg DM)					
Dry matter	693.1	690.1	690.0	702.9	680.3	694.7	663.8	679.6	0.96	0.055	0.08	0.606

**Organic matter**	715.1	716.2	712.2	727.2	701.6	715.6	681.7	700.1	0.89	0.017	0.030	0.678
**Crude protein**	743.8	722.5	750.2	745.2	724.9	729.6	710.9	713.4	0.87	0.012	0.409	0.386
**NDF**	606.0	604.2	571.3	609.1	512.9	538.8	404.3	454.0	1.58	<0.001	0.018	0.418
**Feed conversion**	5.8	6.5	5.5	7.2	5.9	6.7	6.0	7.3	0.48	0.893	<0.001	0.061
**Feed efficiency**	0.2	0.2	0.2	0.1	0.2	0.1	0.2	0.1	0.01	0.748	<0.001	0.141

SE = standard error; L = level of Tifton 85 replacement for *C. argentea* hay in the roughage to replace Tifton 85 hay; C = collection period; L×C = interaction.

As far as the digestibility coefficient of DM, no difference ($P > 0.05$) was observed with Tifton 85 replacement levels for Cratylia hay or collection period. The digestibility coefficients of OM showed a significant ($P < 0.05$) decrease from 715.1 to 681.7 g/kg DM, with increased proportions of Cratylia in the roughage portion of the diet to replace Tifton 85. The same trend was observed on CP, wherein greater proportions of Cratylia decreased ($P < 0.05$) the CP digestibility from 743.8 to 710.9 g/kg DM, from control to the treatment with 100% of Cratylia hay. Regarding NDF, there was also a steady decrease ($P < 0.05$) in digestibility, which ranged from 606 to 404.3 g/kg DM, with increased levels of Cratylia.

In respect to collection periods, the digestibility coefficient of OM had increased ($P < 0.05$) from 712.2 to 727.2, 701.6 to 715.6, and 681.7 to 700.1 g/kg DM at 20%, 40%, and 100% from initial to the final collection period, respectively. The same was observed for NDF, which the digestibility increased ($P < 0.05$) from 571.3 to 609.1, 512.9 to 538.8, 404.3 to 454.0 g/kg DM at 20%, 40%, and 100% from initial to the final collection period, respectively. Nevertheless, no significant differences between collection periods were observed on digestibility coefficients of DM and CP.

The feed conversion (FC) and feed efficiency (FE) did not differ (*P* > 0.05) with Tifton 85 replacement levels for Cratylia's hay. However, FC was greater in the final period (*P* < 0.05), while FE was greater in the initial period.

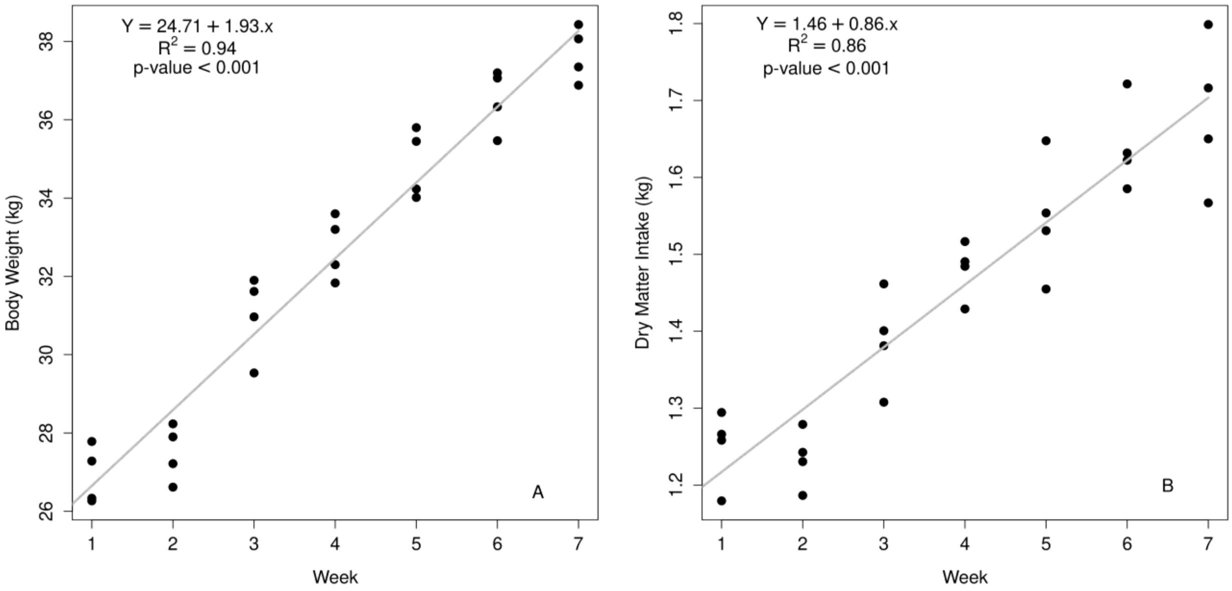

Fig. 1. Body Weight of lambs (A) as a function of weeks of hay feeding and Dry Matter Intake of the lambs (B) as a function of weeks of hay feeding.

Dry matter intake was positively (P<0,01) influenced by weeks of hay feeding, whereas DMI was increased by 0.858 g per animal per week (Fig. 1A). As far as WG, a similar pattern was observed, considering that WG increased 1.93 kg per animal per week (P<0,01) (Fig. 1B).

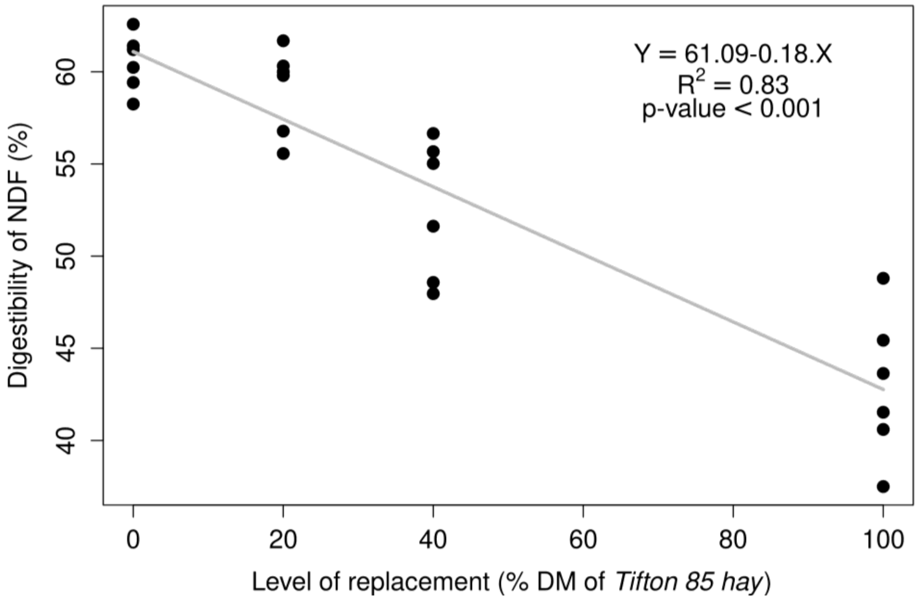

Fig. 2. Digestibility of neutral detergent fiber as a function of Tifton 85 hay (% DM) replacement levels in the diet.

The digestibility coefficient of NDF decreased linearly by 0.18% every week with the Tifton 85 replacement for Cratylia hay (% DM) in the diet (Fig. 2). Therefore, as estimated by the regression equation, the replacement levels of 20, 40, and 100% would cause a decrease on NDF digestibility by 57.42, 53.76, and 42.77%, respectively. Although, this behavior did not negatively influence lamb performance, an accentuated decrease in NDF digestibility was observed at 100% replacement of Tifton 85 with the Cratylia.

Table 7. Nitrogen balance in feedlot Lacaune lambs fed diets containing different levels of *C. argentea* hay replacing Tifton 85 hay at two evaluation times.

Variable	0%		20%		40%		100%			*P*-value		
	Initial	Final	Initial	Final	Initial	Final	Initial	Final	SE	L	C	L×C
N intake (g/day)	40.4	45.3	43.1	52.5	41.9	48.1	43.2	52.6	4.34	0.395	< 0.001	0.537
Fecal N (g/day)	10.4	12.6	10.8	13.4	11.1	13.0	12.4	15.0	1.18	0.294	< 0.001	0.939

**Absorbed N (g/day)**	30.0	32.7	32.3	39.1	30.8	35.1	30.7	37.6	3.22	0.286	< 0.001	0.392
**Urinary N (g/day)**	9.5	14.9	9.3	13.6	8.7	13.8	7.3	12.4	1.72	0.259	< 0.001	0.953
**Retained N (g/day)**	20.8	18.1	23.2	25.7	23.9	23.7	23.4	25.1	2.20	0.258	0.779	0.426
**Retained N/Absorbed N(%)**	68.8	54.7	71.8	65.5	72.0	61.1	75.7	66.8	3.68	0.119	< 0.001	0.651
**Retained N/N intake (%)**	51.5	39.8	53.0	48.8	55.0	49.7	53.8	47.6	3.21	0.255	< 0.001	0.500

SE = standard error; L = level of Tifton 85 replacement levels for *C. argentea* hay in the roughage to replace Tifton 85 hay; C = collection period; L×C = interaction.

Effects of Cratylia hay on nitrogen variables

The level of Tifton 85 replacement for Cratylia in the roughage did not affect any of the nitrogen balance variables (P > 0.05) (Table 7). As far as the collection period, the N intake, fecal N, absorbed N, and urinary N were greater (P < 0.05) in the final collection period. On the other hand, the proportion (%) of Retained to Absorbed N and Retained N to N Intake was greater (P < 0.05) in the initial period. In regard to Retained N (g/day), no changes (P > 0.05) were observed according to collection periods.

Discussion

Lambs' performance on *Cratylia argentea* hay

In most of the measured parameters, the evaluated lambs performed similarly under diets with Tifton 85 replacement for Cratylia hay. Therefore, revealing the capability of Cratylia to be used as animal feed. The use of alternative feedstuff has been a common practice to decrease production cost on livestock systems (Awawdeh, 2011). Producers without the necessary inputs to grow or purchase Tifton 85 hay, may have an alternative with Cratylia, which is a

sustainable feedstuff, since it does not require the use of chemical N fertilizers for high yield and quality biomass production (Andersson et al., 2006).

For the variables of DMI, dry matter digestibility (DMD), FC and FE, Cratylia and Tifton 85 performed the same as well. Dry matter intake was not related to differences in digestibility between the two forage species since the dietary replacement of Tifton 85 for Cratylia did not increase DMD. The particle size uniformity of the diet supplied to the lambs did not allow them to select ingredients, which explains the absence of differences in DMI. Thornton and Minson (1973) reported that the voluntary intake of tropical legumes (when not limited by anti-nutritional factors) is generally greater than that of equally digestible grasses, which may be a consequence of a shorter retention time of legumes in the digestive tract. Likewise, feed particle size may have also influenced the response of the DM digestibility coefficient. The effectiveness of fiber was broken by the particle size of the roughage, so the intestinal passage rate could not reveal possible interactions between the roughages used (Tifton 85 or Cratylia hay). The digestibility coefficient values found in this study are similar to those described by other authors (da Trindade Silva et al., 2021), which included shrub hay and tree legume species as roughage sources in the total diet of feedlot lambs.

The greater DMI with weeks of hay feeding present in this study might be related to an increase in energy required for maintenance, compared to earlier periods of lower BW (Brody, 1945). Cabral et al. (2008) have also found increased DMI and WG in animals with increased weight when evaluating confined lambs. The increased WG supports the findings that Cratylia did not negatively affect the performance on evaluated lambs. The addition of Cratylia

on animal feeds was previously reported to increase (P < 0.05) feed intake from 6.6 to 7.8 and 8.7 kg DM/day, when creole dairy cows were fed sorghum silage alone and supplementation with 2 and 3 kg DM of Cratylia, respectively (Sanchez and Ledin, 2006).

Digestibility

In the matter of OM, CP and NDF digestibility, the increased levels of Cratylia resulted in lower digestibility. Pinzón and Mariño (2010) also reported lower CP and NDF digestibility coefficients in Cratylia hay compared with whole-plant silage or meal. The greater NDF and acid detergent fiber (ADF) contents and lower digestibility of sun-dried forages are attributed to the negative effects of ultraviolet radiation, which can induce the Maillard reaction and thereby increase fiber-bound protein (Van Soest et al., 1991; Dzowela et al., 1995). As an example, nitrogen contents bound to the plant cell wall, which become unavailable to the rumen microbiota (Harper and McNeil, 2015). Moreover, the plants used for hay production were harvested for high DM production (90 days of regrowth) and cut as a whole, including stems and senescent leaves. Therefore, there was no selection for the less lignified material, which may have contributed to greater indigestible fiber content in the diet.

The NDF content of the plants at the time of harvest in this study was similar to the 67.54% and 65.68% found by Herrera and Arguedas (2016) in Cratylia plants harvested at 90 days in the rainy and dry seasons, respectively. It is important to highlight that the lignin present in shrub and tree legumes, as in the case of Cratylia, may be less degradable in the rumen compared with the lignin of grasses (Harper and McNeil, 2015). However, this knowledge is derived

from reports of studies with temperate forages, so the differential action of lignin in tropical legumes is yet to be validated (Castro-Montoya and Dickhoefer, 2020).

Feed conversion and feed efficiency

The greater FC and lower FE in the final period might be attributed to the fact that lambs on the last seven days before finishing were heavier and had a lower growing factor, which limits FE. Pires et al. (2000) suggest that older lambs in the finishing phase tend to have different nutrient demands and composition of weight gain (e.g., more fat than muscle gain), thus, lower FE. Polli et al. (2019) evaluated finishing lambs in lower and higher heat conditions and found a FE decrease of 12.8% and 20.4% on feedlot lambs as the finishing period progressed, respectively. Bowen et al. (2006) implied that FC might be associated with several factors including feed intake, increased age and liveweight of the animal, which explains the findings of this research.

Nitrogen variables

About the increase in N variables at the final collection period (N intake, Fecal N, Absorbed N, Urinary N), it is assumed that greater N content on Cratylia than Tifton 85 (Table 2) justifies the finding. Hess et al. (2004) described greater urinary N excretion in lambs fed diets supplemented with dry leaves of Cratylia, in comparison with the group fed a basal diet with hay of *Urochloa dictyoneura* cv. Lanero. The authors relate this result to the increased urine volume and intake of available water. A similar behavior was observed in the present experiment, which N excretion was greater in the urine than in the feces, especially in the heavier lambs. The greater proportion (%) of Retained to Absorbed N and Retained N to N Intake in the initial period possibly happened due to a greater demand of the growing lambs for proteins. Since part of the N further is

synthesized into proteins by rumen microorganisms (Aquino et al., 2007), to supply the protein demand and increase muscle growth (Prado et al., 2014; Rezaei et al., 2014).

Conclusion

Overall, the replacement of Tifton 85 for Cratylia hay tested demonstrated that lamb performance can be maintained, regardless of replacement level used in the diets. Thus, might be recommended as a food intervention strategy to support livestock production at no cost of chemical fertilizers, since none was used for Cratylia hay production.

The decreased digestibility of NDF, OM and CP on Cratylia's hay may be linked to the method used to preserve the feed and harvest timing. Therefore, further research is expected to identify the parameters that influenced the chemical characteristics of this plant.

Financial Disclosure Statement: The funders had no role in study design, data collection and analysis, decision to publish, or preparation of the manuscript. This work was supported by the National Council of Technological and Scientific Development (CNPq, Brazil #140314/2019-9), Minas Gerais State Agency for Research and Development (FAPEMIG, PPM-00639-18) and the Coordination for the Improvement of Higher Education Personnel (CAPES, Brazil #88887.667823/2022-00).

Availability of data and material: All relevant data are within the manuscript and its Supporting Information files.

Declarations

Ethics approval: all experimental procedures were approved by the Ethics Committee on Animal Use (CEUA) at the Federal University of Minas Gerais (approval no. 192/2020).

Conflict of interest: the authors declare no conflict of interest.

References

Andersson MS, Peters M, Schultze-Kraft R, FRANCO LH, Lascano CE. Phenological, agronomic and forage quality diversity among germplasm accessions of the tropical legume shrub Cratylia argentea. The Journal of Agricultural Science. 2006 Jun;144(3):237-48.

Aquino AA, Botaro BG, Ikeda FD, Rodrigues PH, Martins MD, Santos MV. Efeito de níveis crescentes de ureia na dieta de vacas em lactação sobre a produção e a composição físico-química do leite. Revista Brasileira de Zootecnia. 2007;36:881-7.

Argel PJ and Lascano CE. Cratylia argentea (Desvaux) O. Kuntze: una nueva leguminosa arbustiva para suelos acidos en zonas subhumidas tropicales. Gramineas y leguminosas tropicales... Proyecto IP-5. Circular. Ano 2. 1998.

Association of Official Analytical Chemists (AOAC). Official Methods of Analysis, 15th ed. 1990. Burlington, VA.

Ates S, Cicek H, Bell LW, Norman HC, Mayberry DE, Kassam S, Hannaway DB, Louhaichi M. Sustainable development of smallholder crop-livestock farming in developing countries. InIOP Conference Series: Earth and Environmental Science 2018 Mar 1 (Vol. 142, No. 1, p. 012076). IOP Publishing.

Bowen MK, Ryan MP, Jordan DJ, Beretta V, Kirby RM, Stockman C, McIntyre BL, Rowe JB. Improving sheep feedlot management. International Journal of Sheep and Wool Science. 2006;54(2):27-34.

Brody, S. Bioenergetics and growth: With special reference to the efficiency complex in domestic animals. 1945. Reinhold, New York.

CABRAL LD, SANTOS JW, Zervoudakis JT, Abreu JD, Souza AD, Rodrigues RC. Consumo e eficiência alimentar em cordeiros confinados. Revista Brasileira de Saúde e Produção Animal. 2008 Oct;9(4):703-14.

Campos, FP, Nussio CMB. and Nussio LG, 2004. Métodos de análises de alimentos. Piracicaba, São Paulo.

Castro-Montoya JM, Dickhoefer U. The nutritional value of tropical legume forages fed to ruminants as affected by their growth habit and fed form: A systematic review. Animal Feed Science and Technology. 2020 Nov 1;269:114641.

da Trindade Silva MG, Geraldo Costa M, Campelo Medeiros M, dos Santos Difante G, Sérgio de Azevedo P, Gurgel AL, Emerenciano Neto JV, de Lima Veras EL, Vinhas Ítavo LC. Use of spineless cactus associated with legume hay in the feedlot-finishing of lambs in semi-arid regions. Plos one. 2021 Dec 17;16(12):e0261554.

Dzowela BH, Hove L, Mafongoya PL. Effect of drying method on chemical composition and in vitro digestibility of multi-purpose tree and shrub fodders. Tropical Grasslands. 1995;29(4):263-9.

Fonseca, D.M., Martuscello, J.A., 2010. Plantas Forrageiras. Viçosa, MG: Ed. UFV.

FRANCO AA, de Resende AS, CAMPELLO EF. Importância das leguminosas arbóreas na recuperação de áreas degradadas e na sustentabilidade de sistemas agroflorestais. InEmbrapa Caprinos e Ovinos-Artigo em anais de congresso (ALICE) 2011 Jul 21. In: SEMINÁRIO SISTEMAS AGROFLORESTAIS E DESENVOLVIMENTO SUSTENTÁVEL, 2003, Campo Grande, MS. Palestras... Campo Grande: Embrapa Gado de Corte; Dourados: Embrapa Agropecuária Oeste; Corumbá: Embrapa Pantanal, 2003. 24 f. 1 CD ROM.

Gentil RS, Susin I, Pires AV, Ferreira EM, Mendes CQ, Almeida OC, Queiroz MA. Metabolismo de nutrientes em ovinos alimentados com casca de soja em substituição ao feno de coastcross. Revista brasileira de zootecnia. 2011;40:2835-43.

Gonçalves, G.D., Santos G.T., Cecato, U. Jobim, C.C., Damasceno, J.C., Branco, A.F. and Faria, K.P., 2002. Produção e valor nutritivo de gramíneas do gênero cynodon em diferentes idades ao corte durante o ano. Acta Scientiarum. Animal Sciences, 2008, 24,1163.

Gonçalves GD, dos Santos GT, Cecato U, Jobim CC, Damasceno JC, Branco AF, Faria KP. Produção e valor nutritivo de gramíneas do gênero Cynodon em diferentes idades ao corte durante o ano. Acta Scientiarum. Animal Sciences. 2002;24:1163-74.

Harper KJ, McNeill DM. The role iNDF in the regulation of feed intake and the importance of its assessment in subtropical ruminant systems (the role of iNDF in the regulation of forage intake). Agriculture. 2015 Sep 10;5(3):778-90.

Herrera AM, de Mello AC, de Oliveira Apolinário VX, Dubeux Júnior JC, da Cunha MV, dos Santos MV. Potential of Gliricidia sepium (jacq.) Kunth ex Walp. and Mimosa caesalpiniifolia Benth. in silvopastoral systems intercropped with signalgrass [Urochloa decumbens (Stapf) RD Webster]. Agroforestry Systems. 2021 Aug;95(6):1061-72.

Ibrahim M, Franco M, Pezo DA, Camero A, Araya JL. Promoting intake of Cratylia argentea as a dry season supplement for cattle grazing Hyparrhenia rufa in the subhumid tropics. Agroforestry systems. 2001 Jan;51(2):167-75.

Jaramillo DM, Dubeux Jr JC, Mackowiak C, Sollenberger LE, Santos ER, Garcia L, Ruiz-Moreno M, Silva CS, DiLorenzo N. Annual and perennial peanut species as alternatives to nitrogen fertilizer in bermudagrass hay production systems. Agronomy Journal. 2018 Nov;110(6):2390-9.

Lima, NLL. 2016. Eficiência produtiva em cordeiros classificados pelo Consumo Alimentar Residual (CAR) e Consumo e Ganho Residual (CGR) 132 p. Doctorate thesis, Universidade Federal de Minas Gerais, Belo Horizonte, MG.

Herrera ML, Arguedas EB. Efecto de la frecuencia de corte y la precipitación en el rendimiento de Cratylia argentea orgánica. Nutrición animal tropical. 2016;10(1):24-44.

Luz GA, Gomes SO, de ARAUJO NETO RB, Nascimento MS, LIMA PD. Molecular characterization of accessions of Cratylia argentea (Camaratuba) using ISSR markers.

Maass BL. 1996. Evaluación Agronómica de Cratylia argentea (Desvaux) O. Kuntze en Colombia. In: Pizarro, E.A., Coradin, L. (Eds.), Potencial del Género Cratylia como Leguminosa Forrajera. CIAT, Cali, Colombia, 62–74.

MATRANGOLO WJ, da SILVA IH, ALMEIDA LG, CRUZ SB, MALTA P, GOMES SX. Aspectos ecológicos de Cratylia argentea na região central de Minas Gerais. InEmbrapa Milho e Sorgo-Artigo em anais de congresso (ALICE) 2018. Cadernos de Agroecologia, v. 13, n. 1, jul. 2018.

Maynard LA, Loosli JK, Hintz HF and Warner RG. 1984. Nutrição animal. 3 ed. Rio de Janeiro: Freitas Bastos S.A.

Mehlich A. Mehlich 3 soil test extractant: A modification of Mehlich 2 extractant. Communications in soil science and plant analysis. 1984 Dec 1;15(12):1409-16.

NASA/POWER, The power project, 2022. https://power.larc.nasa.gov/ (accessed 14 July 2022).

National Research Council (NRC). 2007. Nutrient Requirements of Small Ruminants: Sheep, Goats, Cervids and New World Camelids. Washington, DC, 468. National Academy Press. doi.org/10.17226/11654

de Lima Nunes LR, NUNES AM. CAPIM TIFTON 85 SUBMETIDO A DIFERENTES DOSAGENS DE NITROGÊNIO E IDADES DE REBROTA. Revista Científica Rural. 2018 Oct 11;20(2):113-28.

Phelan P, Moloney AP, McGeough EJ, Humphreys J, Bertilsson J, O'Riordan EG, O'Kiely P. Forage legumes for grazing and conserving in ruminant production systems. Critical Reviews in Plant Sciences. 2015 Jun 1;34(1-3):281-326.

Correa Pinzón YT, Niño-Mariño Mariño S. Evaluación de la calidad nutricional de Cratylia argentea sometida a diferentes métodos de conservación, en el Piedemonte llanero. Ciencia Unisalle. https://ciencia.lasalle.edu.co/zootecnia/180 (accessed 6.22.22).

Pires CC, Silva LF, Schlick FE, Guerra DP, Biscaino G, Carneiro RM. Cria e terminação de cordeiros confinados. Ciência Rural. 2000;30:875-80.

Pizarro EA, Carvalho MA, Ramos AK. Introducción y evaluación de leguminosas forrajeras arbustivas en el Cerrado brasileño. En: Potencial del género Cratylia como leguminosa forrajera. 1996. Pizarro EA y Coradin L (eds.). Embrapa, Cenargen, CPAC y CIAT, Memorias Taller sobre Cratylia realizado del 19 al 20 de julio de 1995 en Brasilia, Brasil, 40-49.

Polli VA, Vaz RZ, Carvalho S, Costa PT, de Oliveira Mello R, Restle J, Nigeliskii AF, Silveira ID, Pissinin D. Thermal comfort and performance of feedlot lambs finished in two climatic conditions. Small Ruminant Research. 2019 May 1;174:163-9.

Prado IN, Campo MM, Muela E, Valero MV, Catalan O, Olleta JL, Sañudo C. Effects of castration age, dietary protein level and lysine/methionine ratio on animal performance, carcass and meat quality of Friesian steers intensively reared. Animal. 2014 Sep;8(9):1561-8.

Queiroz LP, Coradin L. Biogeografia de Cratylia e áreas prioritárias para coleta. In: E.A. Pizarro and L. Coradin (eds), Memorias del taller Potencial del género Cratylia como leguminosa forrajera, EMBRAPA/Cenargen/CPAC/CIAT, 19–20, Brasilia, Brasil, 1–12

Queiroz LD. O gênero Cratylia Martius ex Bentham (Leguminosae: Papilionoideae: Phaseoleae): revisão taxonômica e aspectos biológicos. MSc diss., Campinas: Universidade Estadual de Campinas. 1991.

R Core Team. 2021. R: A language and environment for statistical computing. R Foundation for Statistical Computing, Vienna, Austria. https://www.R-project.org/

Ramos AK, PIZARRO E. Algumas informações sobre a produção e o armazenamento de sementes de Cratylia argentea. Embrapa Cerrados. Circular Técnica. 2003.

Rezaei, J., Rouzbehan, Y., Fazaeli, H. and Zahedifar, M., 2014. Effects of substituting amaranth silage for corn silage on intake, growth performance, diet digestibility, microbial protein, nitrogen retention and ruminal fermentation in fattening lambs. Animal Feed Science and Technology, 192, 29-38.

Rezaei J, Rouzbehan Y, Fazaeli H, Zahedifar M. Effects of substituting amaranth silage for corn silage on intake, growth performance, diet digestibility, microbial protein, nitrogen retention and ruminal fermentation in fattening lambs. Animal Feed Science and Technology. 2014 Jun 1;192:29-38.

da Silva ME, de Araújo JV, da Silva JA, de Carvalho LM, das Chagas E, Ribeiro RR. Anthelmintic efficacy of Cratylia argentea (Desv.) Kuntze against the gastrointestinal nematodes of sheep. Semina: Ciências Agrárias. 2017;38(5):3105-12.

Sánchez NR, Ledin I. Effect of feeding different levels of foliage from Cratylia argentea to creole dairy cows on intake, digestibility, milk production and milk composition. Tropical Animal Health and Production. 2006 May; 38:343-51.
Snyder CS, Bruulsema TW, Jensen TL, Fixen PE. Review of greenhouse gas emissions from crop production systems and fertilizer management effects. Agriculture, Ecosystems & Environment. 2009 Oct 1;133(3-4):247-66.
Stagnari F, Maggio A, Galieni A, Pisante M. Multiple benefits of legumes for agriculture sustainability: an overview. Chemical and Biological Technologies in Agriculture. 2017 Dec;4(1):1-3.
Thornton RF, Minson DJ. The relationship between apparent retention time in the rumen, voluntary intake, and apparent digestibility of legume and grass diets in sheep. Australian Journal of Agricultural Research. 1973;24(6):889-98.
Valles-de la Mora B, Castillo-Gallegos E, Alonso-Díaz MÁ, Ocaña-Zavaleta E, Jarillo-Rodríguez J. Live-weight gains of Holstein× Zebu heifers grazing a Cratylia argentea/Toledo-grass (Brachiaria brizantha) association in the Mexican humid tropics. Agroforestry Systems. 2017 Dec;91(6):1057-68.
Van Soest PV, Robertson JB, Lewis BA. Methods for dietary fiber, neutral detergent fiber, and nonstarch polysaccharides in relation to animal nutrition. Journal of dairy science. 1991 Oct 1;74(10):3583-97.
Wanapat M, Totakul P, Viennasay B, Matra M. Sunnhemp (Crotalaria juncea, L.) silage can enrich rumen fermentation process, microbial protein synthesis, and nitrogen utilization efficiency in beef cattle crossbreds. Tropical Animal Health and Production. 2021 Mar;53(1):1-7.
