# Supplementary material for: Could Cratylia argentea replace Tifton 85 hay on growing and finishing lamb diets in tropical areas?
Source: PLoS One. 2023 Dec 11;18(12):e0295510. doi: 10.1371/journal.pone.0295510 (PMC10712867; doi:10.1371/journal.pone.0295510)
Supplement: Attachment — Submitted filename: Response to Reviewers - PONE-D-23-03057.docx [file pone.0295510.s003.docx]

Response to Reviewers

Journal Requirements:

When submitting your revision, we need you to address these additional requirements.

1. Please ensure that your manuscript meets PLOS ONE's style requirements, including those for file naming. The PLOS ONE style templates can be found at 
<https://journals.plos.org/plosone/s/file?id=wjVg/PLOSOne_formatting_sample_main_body.pdf> and 
<https://journals.plos.org/plosone/s/file?id=ba62/PLOSOne_formatting_sample_title_authors_affiliations.pdf>

A: adjusted.

2. In your Methods section, please provide additional information regarding the permits you obtained for the work. Please ensure you have included the full name of the authority that approved the field site access and, if no permits were required, a brief statement explaining why.

A: adjusted.

3. We note that the grant information you provided in the ‘Funding Information’ and ‘Financial Disclosure’ sections do not match.

A: adjusted. Information over the “cover letter” file.

When you resubmit, please ensure that you provide the correct grant numbers for the awards you received for your study in the ‘Funding Information’ section.

4. Thank you for stating the following in the Financial Disclosure Statement of your manuscript:

"The funders had no role in study design, data collection and analysis, decision to publish, or preparation of the manuscript. This work was supported by the National Council of Technological and Scientific Development (CNPq, Brazil #140314/2019-9), Minas Gerais State Agency for Research and Development (FAPEMIG, PPM-00639-18) and the Coordination for the Improvement of Higher Education Personnel (CAPES, Brazil #88887.667823/2022-00). "

We note that you have provided funding information that is not currently declared in your Funding Statement. However, funding information should not appear in the Acknowledgments section or other areas of your manuscript. We will only publish funding information present in the Funding Statement section of the online submission form. 
Please remove any funding-related text from the manuscript and let us know how you would like to update your Funding Statement. Currently, your Funding Statement reads as follows:

"This work was supported by the National Council for Scientific and Technological Development (CNPq, Brazil nº 140314/2019-9), State Agency for Research and Development of Minas Gerais (FAPEMIG, PPM-00639-18) and Coordination for the Improvement of Higher Education Personnel of Education (CAPES, Brazil nº 88887.667823/2022-00). The funders had no role in study design, data collection and analysis, decision to publish, or preparation of the manuscript."

Please include your amended statements within your cover letter; we will change the online submission form on your behalf.

A: Reviewer, the amended information was moved to the Cover Letter, as requested.

5. In your Data Availability statement, you have not specified where the minimal data set underlying the results described in your manuscript can be found. PLOS defines a study's minimal data set as the underlying data used to reach the conclusions drawn in the manuscript and any additional data required to replicate the reported study findings in their entirety. All PLOS journals require that the minimal data set be made fully available. For more information about our data policy, please see <http://journals.plos.org/plosone/s/data-availability>.

Upon re-submitting your revised manuscript, please upload your study’s minimal underlying data set as either Supporting Information files or to a stable, public repository and include the relevant URLs, DOIs, or accession numbers within your revised cover letter. For a list of acceptable repositories, please see <http://journals.plos.org/plosone/s/data-availability#loc-recommended-repositories>. Any potentially identifying patient information must be fully anonymized.

Important: If there are ethical or legal restrictions to sharing your data publicly, please explain these restrictions in detail. Please see our guidelines for more information on what we consider unacceptable restrictions to publicly sharing data: <http://journals.plos.org/plosone/s/data-availability#loc-unacceptable-data-access-restrictions>. Note that it is not acceptable for the authors to be the sole named individuals responsible for ensuring data access.

We will update your Data Availability statement to reflect the information you provide in your cover letter.

6. Your ethics statement should only appear in the Methods section of your manuscript. If your ethics statement is written in any section besides the Methods, please move it to the Methods section and delete it from any other section. Please ensure that your ethics statement is included in your manuscript, as the ethics statement entered into the online submission form will not be published alongside your manuscript.

A: Information adjusted, as requested.

Additional Editor Comments:

Summary: describe in the summary the total period of the experiment. Weight gain between treatments was not shown in the results.

Figure 1A answers this. From the graph, it can be seen that the animals gained weight at a rate of 1.93 kg per week, which gives an average daily gain of (1.93/7 = 275 grams/day).

L70 to 73: Please insert in the sentence the justification for why it would be more suitable for small production systems.

Authors decided to remove this sentence.

The introduction is too long.

Adjusted, as requested.

Suggestion: text from L108 to 123 can be used in the discussion and removed from the introduction.

Indeed. Thanks for the suggestion.

Authors need to be careful in claiming that Cratylia hay can replace 100 % Tifton hay due to the value of nutrients found in Tifton hay that was used in the experiment.

M&M: Why do a digestibility test at the beginning and another at the end?

As this is a new food, we sought to evaluate the performance of the animals at different times during the experiment. That is, growing animals and animals already finishing.

L 150: Could you please verify that this FDN value is correct? It seems very low to me than normally found for this food.

The Neutral Fiber Detergent (NFD) values are consistent with those found in other studies.

Gama, T. D. C. M., Zago, V. C. P. Nicodemo, M. L. F., Laura, V. A., Volpe, E., & Morais, D.G. (2009). Composição bromatológica, digestibilidade in vitro e produção de biomassa de leguminosas forrageiras lenhosas cultivadas em solo arenoso. Revista Brasileira de Saúde e Produção Animal, 10(3), 560-572.

Montero-Durán, E., Rojas-Bourrillon, A., & López-Herrera, M. (2021). Sustitución de Cratylia argentea y Erythrina poeppigiana por guineo cuadrado en ensilados. Nutrición Animal Tropical, 15(2), 123-146. <https://doi.org/10.15517/nat.v15i2.48818>

ÁLVAREZ CARRILLO, F.; FERNANDO CASANOVES; CUELLAR MEDINA, Y.; ORTIZ MENESES, J. F.; BALANTA MARTINEZ, V. J.; CELIS PARRA, G. A. Nutritional quality of Piptocoma discolor and Cratylia argentea as a non-timber forest products for animal feed in the Caquetá province. Journal of Agriculture and Environment for International Development (JAEID), [S. l.], v. 116, n. 2, p. 109–120, 2023. DOI: 10.36253/jaeid-13102. <https://www.jaeid.it/index.php/jaeid/article/view/13102>

Table 3: Data can be described in the text. There is no need for them to be transcribed into a table.

Adjusted.

L283 to 285: I think the authors are misinterpreting the results. In the way the results were arranged, when there is the significance of L (treatment) as only one P was generated, it means that the results used for the statistics were the data set of both periods (715.1 and 716.2) which would be an average close to 715.65 for 0 and 690.9 for 100% treatment (681.7 and 700.1). If the P is specific for the initial period, then the authors must provide the other probabilities and indicate with letters where there was a difference.

L290 to 292: display different letters when there is a statistical difference between means. The table should be self-explanatory and show all existing results.
Table 5. Data are hard to follow. It would be interesting for the authors to provide a more didactic disposition of the results.

Tables. Present two decimal places after the point to better understand the variability of the data. The standard error of the mean is two decimal places.

Adjusted.

Statistics: Did the authors test the strength of the experiment? From the averages found in most of the variables and the respective standard errors, it is doubtful that there was no difference because the experiment was not strong enough to obtain differences due to the low repetition of animals per treatment.

To detect small differences between the means, for data that have a high standard deviation, a high number of repetitions would be necessary, that is, a very large number of animals, which could make timely research unfeasible, as approved by the ethics council.

L318 to 320. The authors describe that the animals were weighed weekly and do not present any weight or weight gain data from the treatments. Only in time. Please present the results with the respective standard errors of the mean and probabilities. It is also important to better visualize the efficiency results.

Added table with this information

Results: The authors have the digestibility coefficients and did not present the nutrient intake. Why? If we do the DMI calculations, animals with 100 g of C. argentea hay ingested 300 g more food per day compared to 100 g of Tifton. Is it worth it, isn't it worth it for food costs? This would be an interesting part for the authors to discuss in the 'Discussion' section.

Indeed. Thanks for the suggestion.

The objective of the present study is to evaluate Cratylia as a new food that can be introduced into the market for feeding ruminants, however, there is no pricing yet. However, the productive seasonality of tropical forage grasses, such as Tifton-85 grass, requires investments in infrastructure and machinery, which consequently reduce the net profitability of the production system. Therefore, the use of Cratylia can be an interesting alternative, especially where conditions for the cultivation of other forage species may be limited.

Discussion: The discussion needs to be revised and improved. In most of the text, the authors discuss the differences in time due to the growth of the animals and compare them with the results of other authors. They do not bring a discussion that advances knowledge about the purpose of the study.

The authors affirm the high content of lignin in the hay of C. argentea but do not present the results of this component in the work. I think that to use this discussion it would be interesting to present the results of lignin and/or to present average data on the amount of this component of other authors who used this food in their research.

Adjusted.

L406: The fact that the greater the amount of ingested protein causes more water consumption is explained by physiology. To excrete nitrogen the organism needs to do it together with water. Please review physiology studies.

Indeed. Thanks for the suggestion.

Conclusion: The authors need to reflect a little more on the results obtained to conclude the complete replacement of C. argentea hay by Tifton hay, given the high variability of the results and the low repetition of experimental units. Mainly by the results of digestibility coefficients found. Unless even the food has worse digestibility, and with the additional cost of offering this food to obtain the same weight gain, it is economically compensated for the producer.

Conclusion: Please remove the last paragraph from the conclusion because nutrient parameters in different hays harvested at different times were not studied.

Adjusted.

Please get the references right.

Reviewers' comments:
Reviewer's Responses to Questions

**Comments to the Author**

1. Is the manuscript technically sound, and do the data support the conclusions?

The manuscript must describe a technically sound piece of scientific research with data that supports the conclusions. Experiments must have been conducted rigorously, with appropriate controls, replication, and sample sizes. The conclusions must be drawn appropriately based on the data presented.

Reviewer #1: Yes

Reviewer #2: Yes

2. Has the statistical analysis been performed appropriately and rigorously?

Reviewer #1: Yes

Reviewer #2: Yes

3. Have the authors made all data underlying the findings in their manuscript fully available?

The [PLOS Data policy](http://www.plosone.org/static/policies.action#sharing) requires authors to make all data underlying the findings described in their manuscript fully available without restriction, with rare exception (please refer to the Data Availability Statement in the manuscript PDF file). The data should be provided as part of the manuscript or its supporting information, or deposited to a public repository. For example, in addition to summary statistics, the data points behind means, medians and variance measures should be available. If there are restrictions on publicly sharing data—e.g. participant privacy or use of data from a third party—those must be specified.

Reviewer #1: Yes

Reviewer #2: Yes

4. Is the manuscript presented in an intelligible fashion and written in standard English?

PLOS ONE does not copyedit accepted manuscripts, so the language in submitted articles must be clear, correct, and unambiguous. Any typographical or grammatical errors should be corrected at revision, so please note any specific errors here.

Reviewer #1: Yes

Reviewer #2: Yes

5. Review Comments to the Author

Please use the space provided to explain your answers to the questions above. You may also include additional comments for the author, including concerns about dual publication, research ethics, or publication ethics. (Please upload your review as an attachment if it exceeds 20,000 characters)

Reviewer #1: “Could Cratylia argentea replace Tifton 85 hay on finishing lamb diets in tropical areas?” it is an original work and brings a good proposal for an alternative replacement to a food that is already consolidated in the diet of ruminants in Brazil. The work is adequate and offers sufficient subsidies for publication in Plos One, provided that the authors consider all the suggestions made in the manuscript. The title begins with a question, based on the findings, the authors are expected to answer in the **conclusion clearly and to recommend the replacement level**.

Adjusted in the authors' best discretion

Reviewer #2: General points about the manuscript: The manuscript brings interesting information regarding evaluation of alternative feedstuff. The manuscript is very well vritten, with sufficicient information, but there are some especifics issues that must be reviewed.

Line 142 to 148: The paragraph has significant wordiness and awkward phraseology. Please try to make the paragraph more explanatory. Rewriter. It is confusing.

Adjusted in the authors' best discretion.

Line 174 – Drums is the best word to use? Check it.

Adjusted.

Line 176 - …November 2020 and lasted January 2020. Is the year “January 2020” correct?

Adjusted.

The lambs stay 64 days in an individual metabolic cages?

Yes.

The word “orts” is the best word to the context?

Yes, it is.
