# Supplementary material for: Could Cratylia argentea replace Tifton 85 hay on growing and finishing lamb diets in tropical areas?
Source: PLoS One. 2023 Dec 11;18(12):e0295510. doi: 10.1371/journal.pone.0295510 (PMC10712867; doi:10.1371/journal.pone.0295510)
Supplement: Attachment — Submitted filename: Response to Reviewers 2 - PONE-D-23-03057 - 2023-11-07.docx [file pone.0295510.s004.docx]

Please review your reference list to ensure that it is complete and correct. If you have cited papers that have been retracted, please include the rationale for doing so in the manuscript text or remove these references and replace them with relevant current references. Any changes to the reference list should be mentioned in the rebuttal letter that accompanies your revised manuscript. If you need to cite a retracted article, indicate the article’s retracted status in the References list and include a citation and full reference for the retraction notice.

Additional Editor Comments:

The authors sent the first version without line numbers, which made the manuscript review process very difficult. Please insert line numbers in the next revision.

**Adjusted.**

Be careful when sending files. They include comments from the authors.

**Thanks!**

Please correct the punctuation in the introduction. By changing the names of the authors to numbers, the text was distorted. There are also other parts of the text that need to be revised for spacing and punctuation.

**Information adjusted.**

Abstract: The objective of this study was to evaluate the performance of 'growing' and finishing Lacaune lambs fed Cratylia argentea hay as an alternative to Tifton 85 (Cynodon spp).

**I have changed from “finishing” to growing and finishing throughout the manuscript, as requested.**

Keywords suggestion: forage conservation, forage digestibility, ruminant feeding, shrub legume, small ruminant performance

**Suggestion taken!**

In the Results section, please present the real probabilities found for the respective variables and not less than 0.05.

**Thanks for the suggestion, but we would rather keep it this way. The numbers are too big to fit in the table. In case you need it, I can provide it for you over the next response to reviewers’ file.**

Please provide ADF values for Tifton hay.

**Unfortunately, we do not have those values.**

It was requested that the lignin values of the respective hays be presented, mainly from Cratylia argentea as there was discussion about this component, the authors responded that it was adjusted but I did not find it in the final version of the manuscript.

**Unfortunately, we did not evaluate the lignin content. The lignin is only cited in the text, based on plant physiological conclusions of other authors.**

Table 5: if there was no interaction between treatment and period, please remove the P values for LxC.

**Information removed, as requested.**

Table 5 = table 6?????

**Information adjusted.**

Table 7: Please correct. The value presented as BW does not match the weight of the animals presented at the beginning of the M&M of 21.5 ± 3.38 kg. I think the authors must be presenting weight gain here. But I ask the authors' attention because in the answer given to the questions it was written that the weight gain was 275g/day and the table says 250 g/day.

**Information adjusted. Thanks for the heads up. The values on table 7 were adjusted. The correct was 275 g/d, since the ADG was based on the weekly gain of 1.93 kg.**

Favor verificar os dados apresentados na tabela 5 e 6.

Table 5 0% 20% 40% 100%

DMI 1.50 1.58 1.47 1.64

Digestibility 692.72 697.54 688.64 672.84

Table 6

" Actual MS intake 1.035 1.09 0.99 1.10"

Table 7 1.50 1.60 1.51 1.66

**After a discussion with other authors, we concluded that table 6 was redundant. Thus, it was removed. Thanks!**

There are two tables 7. In the second table 7, as there was no interaction between LxC, please remove it from the table and only describe in the text that there was no interaction for these variables.

**Suggestion taken.**

Please present this nitrogen data in the same format shown in table 6.

Discussion: the 100% *C. argentea* treatment had the worst N retention ratio. This means greater nitrogen excretion into the soil. And this is not desirable. I think the authors should take this into account in the discussion and conclusion.

**Very well observed. Thanks a lot. We have made some arrangements. Find it over the lines 402-411.**

Conclusion: digestibility results were worse for C. argentea hay when at 100% replacement. We can see that numerically the conversion efficiency values were worse for this treatment (100%). No difference was detected because variability is high. Absence of evidence is not evidence of absence. I suggest the authors evaluate their conclusion in suggesting that C. argentea hay can replace Tifton hay by 100%.

**Good catch. Information adjusted. Find it over the lines 415-420.**

General suggestions: consider using a greater number of repetitions per treatment in future experiments. Six is a low number for performance experiments due to the variability of the respective variable.

**Thanks for the suggestion.**
