# Supplementary material for: Could Cratylia argentea replace Tifton 85 hay on growing and finishing lamb diets in tropical areas?
Source: PLoS One. 2023 Dec 11;18(12):e0295510. doi: 10.1371/journal.pone.0295510 (PMC10712867; doi:10.1371/journal.pone.0295510)
Supplement: Attachment — Submitted filename: Response to Reviewers 3 - PONE-D-23-03057 - 2023-11-16.docx [file pone.0295510.s005.docx]

Please review your reference list to ensure that it is complete and correct. If you have cited papers that have been retracted, please include the rationale for doing so in the manuscript text or remove these references and replace them with relevant current references. Any changes to the reference list should be mentioned in the rebuttal letter that accompanies your revised manuscript. If you need to cite a retracted article, indicate the article’s retracted status in the References list and include a citation and full reference for the retraction notice.

Additional Editor Comments:

Please, when sending the revised manuscript again, send it with the accepted considerations highlighted in yellow and not in the 'control changes' review mode. This makes the review work very difficult.

**Just did.**

Please present this nitrogen data (table 6) in the same format shown in table 5. It was requested in the previous opinion.

**Adjusted. Find the table over the line 310 and highlighted in yellow as requested.**

In the nitrogen table 6 there is a number marked (3) as excluded which I think was not meant to be excluded (13.83). Please check.

**Indeed! That was probably deleted by mistake.**
